# Supplementary material for: Nutrition-Related Information on Instagram: A Content Analysis of Posts by Popular Australian Accounts
Source: Nutrients. 2023 May 16;15(10):2332. doi: 10.3390/nu15102332 (PMC10223443; doi:10.3390/nu15102332)
Supplement: Supplementary file 1 [file nutrients-15-02332-s001.zip › nutrients-2368668-supplementary.pdf]

Table S1. Standards for Reporting Qualitative Research checklist

No.	Topic	Item	Page number in manuscript
**Title and abstract**			
S1	Title	Concise description of the nature and topic of the study. Identifying the study as qualitative or indicating the approach (e.g., ethnography, grounded theory) or data collection methods (e.g., interview, focus group) is recommended.	1
S2	Abstract	Summary of key elements of the study using the abstract format of the intended publication; typically includes background, purpose, methods, results, and conclusions.	1
**Introduction**			
S3	Problem formulation	Description and significance of the problem/phenomenon studied; review of relevant theory and empirical work; problem statement.	1-2
S4	Purpose or research question	Purpose of the study and specific objectives or questions.	2
**Methods**			
S5	Qualitative approach and research paradigm	Qualitative approach (e.g., ethnography, grounded theory, case study, phenomenology, narrative research) and guiding theory if appropriate; identifying the research paradigm (e.g., post-positivist, constructivist/interpretivist) is also recommended; rationale.	2
S6	Researcher characteristics and reflexivity	Researchers' characteristics that may influence the research, including personal attributes, qualifications/experience, relationship with participants, assumptions, and/or presuppositions; potential or actual interaction between researchers' characteristics and the research questions, approach, methods, results and/or transferability.	2
S7	Context	Setting/site and salient contextual factors; rationale.	3
S8	Sampling strategy	How and why research participants, documents, or events were selected; criteria for deciding when no further sampling was necessary (e.g., sampling saturation); rationale.	3
S9	Ethical issues pertaining to human subjects	Documentation of approval by an appropriate ethics review board and participant consent, or explanation for lack thereof; other confidentiality and data security issues.	2-3
S10	Data collection methods	Types of data collected; details of data collection procedures including (as appropriate) start and stop dates of data collection and	3

		analysis, iterative process, triangulation of sources/methods, and modification of procedures in response to evolving study findings; rationale.	
S11	Data collection instruments and technologies	Description of instruments (e.g., interview guides, questionnaires) and devices (e.g., audio recorders) used for data collection; if/how the instrument(s) changed over the course of the study.	3
S12	Units of study	Number and relevant characteristics of participants, documents, or events included in the study; level of participation.	3
S13	Data processing	Methods for processing data prior to and during analysis, including transcription, data entry, data management and security, verification of data integrity, data coding and anonymization / de-identification of excerpts.	3
S14	Data analysis	Process by which inferences, themes, etc. were identified and developed, including the researchers involved in data analysis; usually references a specific paradigm or approach; rationale.	4-5
S15	Techniques to enhance trustworthiness	Techniques to enhance trustworthiness and credibility of data analysis, (e.g., member checking, triangulation, audit trail); rationale.	3-5
**Results/findings**			
S16	Synthesis and interpretation	Main findings (e.g., interpretations, inferences, and themes); might include development of a theory or model, or integration with prior research or theory.	5
S17	Links to empirical data	Evidence (e.g., quotes, field notes, text excerpts, photographs) to substantiate analytic findings.	5-12
**Discussion**			
S18	Integration with prior work, implications, transferability, and contribution(s) to the field	Short summary of main findings, explanation of how findings and conclusions connect to, support, elaborate on, or challenge conclusions of earlier scholarship; discussion of scope of application/generalizability; identification of unique contribution(s) to scholarship in a discipline or field.	12-15
S19	Limitations	Trustworthiness and limitations of findings	14-15
**Other**			
S20	Conflicts of interest	Potential sources of influence or perceived influence on study conduct and conclusions; how these were managed.	16
S21	Funding	Sources of funding and other support; role of funders in data collection, interpretation, and reporting.	16

Table S2. Description of Instagram account categories

Category	Description
Lifestyle influencer	Accounts that were held by one individual who primarily posted about their daily life and routine.
Fitness/coaching influencer	Accounts that were held by one individual who primarily posted about exercise and sports nutrition. Accountholders often self-identified as a coach.
Brand	Accounts that were in the name of a brand or company, for example, a supplement company.
Recipes/meal ideas	Accounts that primarily posted recipes or meal ideas.
Nutritionist or dietitian influencer	Accounts that were held by one individual who self-identified as a nutritionist or a dietitian.
Media entity	Accounts that were run on behalf of a media entity such as a television show or magazine.
